# The Effects of High-Intensity Interval Training on Exercise Capacity and Prognosis in Heart Failure and Coronary Artery Disease: A Systematic Review and Meta-Analysis

**DOI:** 10.1155/2022/4273809

**Published:** 2022-06-09

**Authors:** Cuihua Wang, Jun Xing, Baoli Zhao, Yahui Wang, Lizhuang Zhang, Yebo Wang, Mingqi Zheng, Gang Liu

**Affiliations:** ^1^Rehabilitation Medicine Department, The First Hospital of Hebei Medical University, Shijiazhuang, Hebei, China; ^2^Department of Cardiology, The First Hospital of Hebei Medical University, Shijiazhuang, Hebei, China

## Abstract

**Objective:**

The purpose of this study is to compare the effects of high-intensity interval training (HIIT) versus moderate-intensity continuous training (MICT) on exercise capacity and several prognostic markers in patients with coronary artery disease (CAD) and heart failure (HF).

**Methods:**

This systematic review is registered on the INPLASY website (number: INPLASY202080112). We conducted a comprehensive search in eight databases of literature before September 13, 2019. Trials comparing HIIT and MICT in participants with CAD or HF aged 52–78 years were included. Exercise capacity (peak oxygen consumption (peak VO_2_)) and prognostic markers, such as the anaerobic threshold (AT), minute ventilation/carbon dioxide production (VE/VCO_2_) slope, left ventricular ejection fraction (LVEF), and prognostic value of the predicted VO_2_ max per cent (the predicted VO_2_ peak (%)) were examined.

**Results:**

A total of 15 studies were included comprising 664 patients, 50% of which were male, with an average age of 60.3 ± 13.2 years. For patients with CAD, HIIT significantly improved peak VO_2_ values (95% CI 0.7 to 2.11) compared with MICT, but peak VO_2_ values in patients with HF did not seem to change. For training lasting less than eight weeks, HIIT significantly improved peak VO_2_ values (95% CI 0.70 to 2.10), while HIIT lasting 12 weeks or longer resulted in a modestly increased peak VO_2_ value (95% CI 0.31 to 5.31). High-intensity interval training significantly increased the AT when compared with MICT (95% CI 0.50 to 1.48). High-intensity interval training also caused a moderate increase in LVEF (95% CI 0.55 to 5.71) but did not have a significant effect on the VE/VCO_2_ slope (95% CI −2.32 to 0.98) or the predicted VO_2_ peak (95% CI −2.54 to 9.59) compared with MICT.

**Conclusions:**

High-intensity interval training is an effective therapy for improving peak VO_2_ values in patients with CAD. High-intensity interval training in the early stage (eight weeks or fewer) is superior to MICT. Finally, HIIT significantly improved prognostic markers, including the AT and LVEF in patients with CAD and HF.

## 1. Introduction

It has been well established that cardiovascular diseases are a leading cause of disability and death worldwide, and among them, the most common are coronary artery disease (CAD) [1] and heart failure (HF) [2]. Despite great progress in diagnostic and therapeutic strategies for CAD and HF over the past few decades, the prognosis remains poor, placing a high burden on healthcare systems worldwide.

Cardiac rehabilitation is an important component of secondary prevention of cardiovascular diseases. Some evidence suggests that cardiac rehabilitation can reduce the mortality risk and improve the prognosis among patients with cardiovascular diseases [3, 4], and one of the most indispensable elements of such an intervention is participation in exercise training [5]. Although exercise training has positive effects on CAD and HF, such as increasing exercise capacity and improving left ventricular function [6, 7], the most effective treatment options are likely dependent upon the type of exercise undertaken [8]. Currently, an increasing number of studies show high-intensity interval training (HIIT) may elicit greater benefits than moderate-intensity continuous training (MICT) in terms of the quality of life and exercise capacity (as measured by the peak oxygen consumption (peak VO_2_) value) of patients with CAD [9] and HF [10]. However, HIIT remains a controversial alternative exercise modality due to its feasibility, safety and long-term adherence. Due to a lack of a systematic review, the objective of our meta-analysis was to compare the effects of HIIT and MICT on the exercise capacity in patients with CAD and HF. The secondary aims were to compare the effects of HIIT and MICT on the prognostic characteristics of the anaerobic threshold (AT), left ventricular ejection fraction (LVEF), the minute ventilation/carbon dioxide production (VE/VCO_2_) slope and the predicted VO_2_ peak (%) in a group of patients with CAD and HF.

## 2. Methods

### 2.1. Data Sources

We conducted a comprehensive literature search in the following eight electronic databases from database inception through September 13, 2019: PubMed, Cochrane Library, Embase, ClinicalTrials, Web of Science, Wanfang Data, China Biology Medicine databases, and the China Knowledge Network. The Medical Subject Heading database was employed to establish all related articles on HIIT and cardiac rehabilitation. This systematic review was registered on the INPLASY website (number: INPLASY202080112). The organisation which was responsible for the integrity and conduct of the report was the First Hospital of Hebei Medical University. The protocol for this systematic review was registered and is available on the INPLASY (http://inplasy.com/) website (10.37766/inplasy00000000).

The following keywords were used as the search terms: ‘rehabilitation/rehabilitations, cardiac'; ‘cardiovascular rehabilitation/rehabilitations'; ‘high-intensity interval trainings'; ‘exercises/exercise, high-intensity intermittent'; ‘sprint interval training' and their related terms (as shown in Table 1). The reference lists of all included studies were also searched to identify other appropriate studies. No language limitations were imposed.

### 2.2. Study Selection

After removing all duplicates, two researchers (Yahui Wang and Yebo Wang) independently reviewed all titles and abstracts to identify potentially eligible studies that primarily reported the effect of HIIT on the peak VO_2_ value in patients with CAD and HF. This meta-analysis was conducted in accordance with the guidelines of the Preferred Reporting Items for Systematic Review and Meta-analyses [11]. The selection criteria were as follows: (1) included adult patients (aged ≥18 years) with CAD and HF (LVEF < 45%); (2) measured at the least peak oxygen uptake (peak VO_2_); and (3) included a comparator group that completed MICT (matched to HIIT). The exclusion criteria were as follows: (1) nonfull articles and nonrandomised controlled trials; (2) missing or insufficient original data; (3) EF was not reported in patients with HF (LVEF > 45%); and (4) studies did not meet the Weston et al. [12] inclusion criteria for both groups. Other outcomes of interest included the AT, VE/VCO_2_ slope, predicted VO_2_ peak, and LVEF.

### 2.3. Data Synthesis and Analysis

Two researchers (Jun Xing and Lizhuang Zhang) independently extracted all relevant data and stored them in a database for analysis; the data included baseline and postintervention mean ± standard deviation and the number of participants in each group. The mean difference and 95% confidence intervals (95% CI) for each parameter were calculated using the Review Manager version 5.3 software. If necessary, study authors were contacted for missing values. We used the Cochrane Collaboration's tool to assess risk of bias in the included trials. Heterogeneity was quantified using the *I*^2^ statistic; *I*^2^ values of 25%, 50%, and 75% represented low, moderate, and high heterogeneity, respectively. An *I*^2^ > 50% indicated significant heterogeneity, and we used the random effects model for meta-analyses; otherwise, a fixed effects model was used [13]. Subgroup and sensitivity analyses were conducted to identify possible sources of heterogeneity by disease categories (CAD versus HF) and the duration of the exercise programmes (<8 weeks, 8–12 weeks, and ≥12 weeks). We used the Begg adjusted rank correlation test [14] and Egger's regression asymmetry test [15] to assess publication bias when there were 10 or more studies. No evidence of publication bias was found. The analyses were performed using the STATA software (v 14.0, StataCorp LLC).

## 3. Results

### 3.1. Study Screening, Selection, and Evaluation

#### 3.1.1. Study Selection

**Figure 1:**
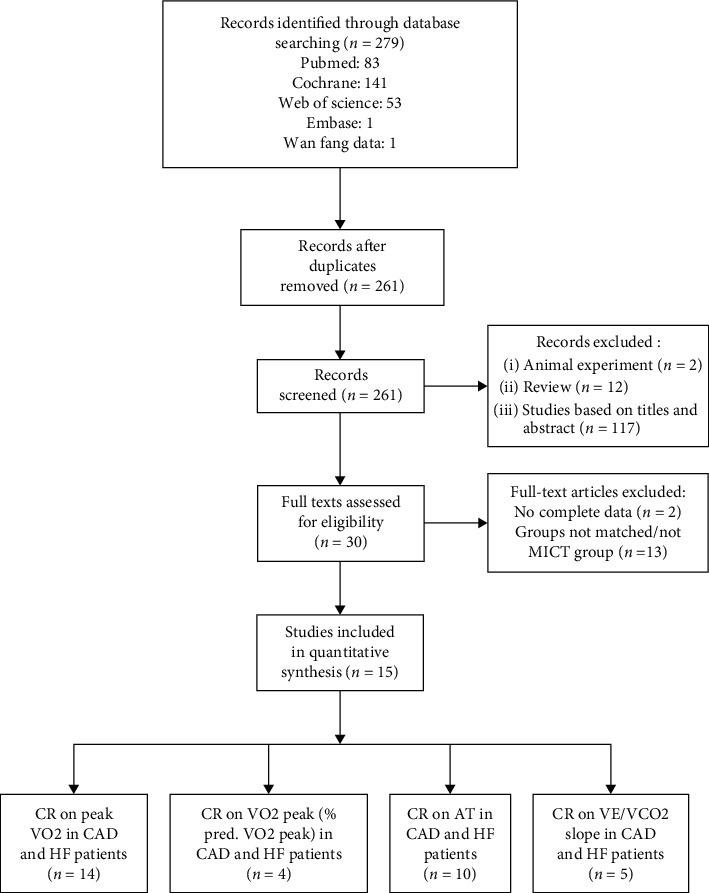
Systematic review process. CAD: coronary artery disease; HF: heart failure.

After a primary search of eight databases, 279 articles were obtained. The subject of 18 papers overlapped with other publications. A total of 12 studies were reviewed and two studies were found to be animal experiments. Another 117 studies were excluded according to the title and abstract. Thirty full manuscripts were reviewed, of which two did not provide complete data for this meta-analysis and 13 were not matched for the MICT group. Finally, 14 studies were adopted to analyse the peak VO_2_ values [16–29], 10 studies were adopted to analyse the AT [16, 18–20, 23, 25, 26, 28–30], five studies were adopted to analyse the VE/VCO_2_ slope [17–19, 24, 30], and four studies each were adopted to analyse the predicted VO_2_ peak [17, 18, 24, 25] and LVEF in patients with CAD and HF [16–18, 22]. The study selection process is summarised in Figure 1. In this study, there were 664 patients with an average age of 60.3 ± 13.2 years and 502 male patients (79.9%).

#### 3.1.2. Studies Included in the Systematic Review

**Figure 2:**
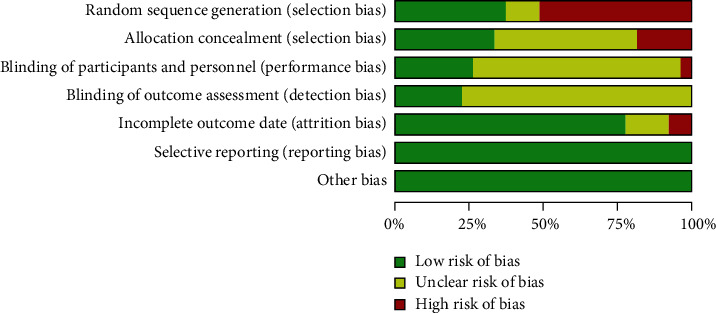
Risk of bias assessment.

The 15 unique randomised controlled trials (Table 2) included eight patients with CAD [19–21, 25, 27–30] and seven patients with clinically stable HF with reduced ejection fraction [1, 16, 17, 22–24, 26]. The 15 studies examined the base case results of 664 patients (50% were male); 338 completed a HIIT intervention (78.7% were male with a mean age of 57.1 years), and 326 completed an MICT intervention (81.5% were male with a mean age of 61.3 years). The duration of training varied between the studies, from 3.5 weeks to 16 weeks. Table 2 shows participant demographics, and Table 3 outlines intervention characteristics. Although all studies were randomised, only six (40%) described random sequence generation, four reported allocation concealment, three trials were double-blinded, and three trials (20%) adequately described subject withdrawals or dropouts. Accordingly, almost all the examined studies had a high risk of bias for sample selection (Figure 2). The exercise modalities consisted primarily of cycle ergometer use (eight studies [17, 18, 20, 21, 25, 26, 28, 29]) followed by treadmill running (three studies [16, 19, 27]); however, three studies focused on cycle ergometer use or treadmill running [22–24] and one study focused on treadmill running or climbing stairs [30].

#### 3.1.3. Quantitative Data Synthesis

**Figure 3:**
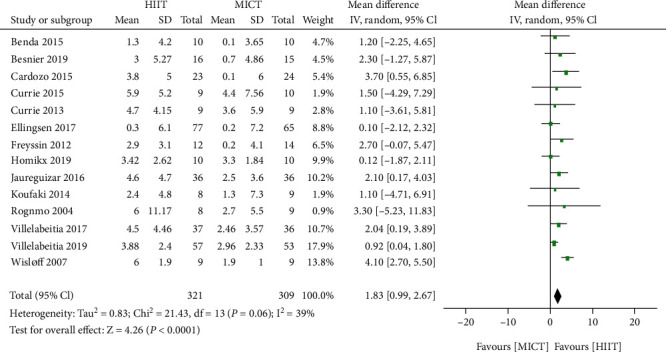
Forest plots of the effect of HIIT and MICT on peak VO_2_ for patients with CAD and HF. HIIT: high-intensity interval training; MICT: moderate-intensity continuous training; peak VO_2_: peak oxygen consumption; CAD: coronary artery disease; HF: heart failure; SD: standard deviation; CI: confidence interval.

**Figure 4:**
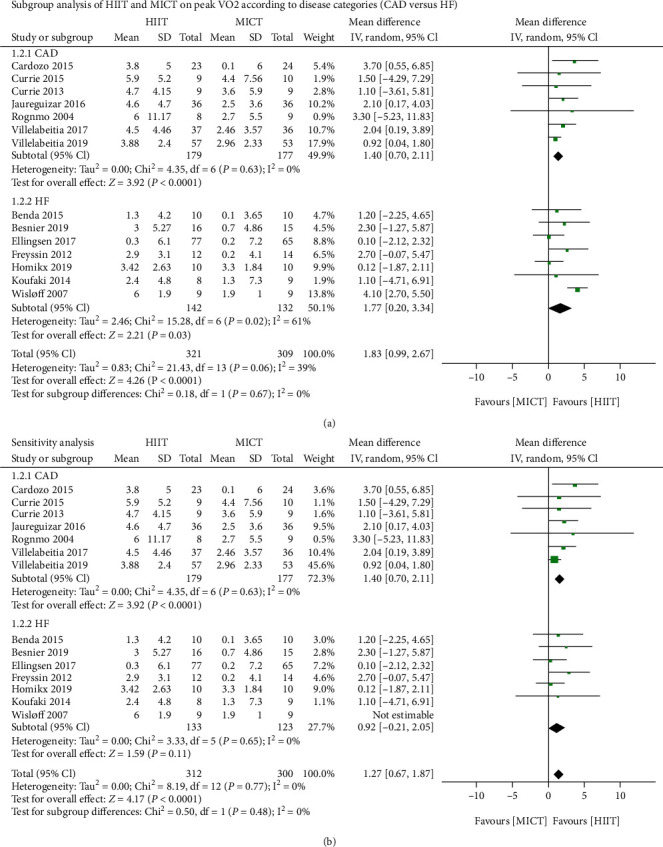
(a) Subgroup analysis of HIIT and MICT on peak VO_2_ according to disease categories (CAD versus HF); (b) sensitivity analysis. HIIT: high-intensity interval training; MICT: moderate-intensity continuous training; peak VO_2_: peak oxygen consumption; CAD: coronary artery disease; HF: heart failure; SD: standard deviation; CI: confidence interval.

Compared with MICT, HIIT significantly improved the peak VO_2_ value (14 trials; 650 patients; mean difference of 1.83 mL/kg/min, 95% CI 0.99 to 2.67 mL/kg/min, *P* < 0.0001; Figure 3); however, there was a relatively large heterogeneity between the trials (*I*^2^ = 39.0%). Accordingly, further analysis based on disease categories (CAD versus HF) revealed that HIIT in the CAD group caused significantly increased peak VO_2_ values (seven trials [19–21, 25, 27–29]; 360 patients; mean difference of 1.4 mL/kg/min, 95% CI 0.7 to 2.11 mL/kg/min, *I*^2^ = 0%, *P* < 0.0001), while in the HF group, it resulted in modestly increased peak VO_2_ values (seven trials [16–18, 22–24, 26]; 290 patients; mean difference of 1.77 mL/kg/min, 95% CI 0.20 to 3.34 mL/kg/min, *P* = 0.03; Figure 4(a)). The heterogeneity test (*P* = 0.02, *I*^2^ = 61%) showed there was notable heterogeneity in the studies. As can be seen from the heterogeneity forest plots, one study [16] was a likely source of heterogeneity. After exclusion of this study, the heterogeneity was significantly reduced. Interestingly, we found no statistically significant difference in the mean difference and 95% CI of the heterogeneity test (*P* = 0.65, *I*^2^ = 0%; mean difference of 0.92 mL/kg/min, 95% CI −0.21 to 2.05 mL/kg/min, *P* = 0.11; Figure 4(b)).

**Figure 5:**
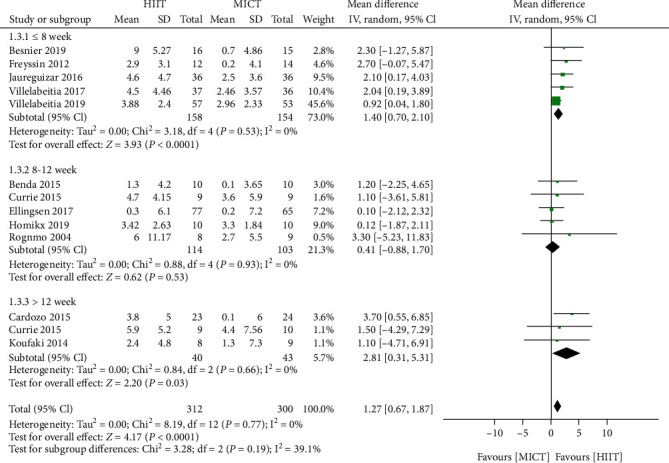
Subgroup analysis of HIIT and MICT on peak VO_2_ according to training duration (≤8weeks, 8-12weeks, and ≥12weeks). HIIT: high-intensity interval training; MICT: moderate-intensity continuous training; peak VO_2_: peak oxygen consumption; CAD: coronary artery disease; HF: heart failure; SD: standard deviation; CI: confidence interval.

Further analysis according to the exercise training programme length (<8 weeks, 8–12 weeks, and ≥12 weeks) revealed that HIIT lasting eight weeks or fewer significantly increased the peak VO_2_ value (five trials [18, 23, 28–30]; 312 patients; mean difference of 1.40 mL/kg/min, 95% CI 0.70 to 2.10 mL/kg/min; *I*^2^ = 0%), while HIIT lasting 12 weeks or more resulted in a modestly increased peak VO_2_ value (three trials [19, 21, 26]; 99 patients; mean difference of 2.81 mL/kg/min, 95% CI 0.31 to 5.31 mL/kg/min; *I*^2^ = 0%); however, during weeks 8–12 of exercise training, MICT and HIIT were not different (five trials [17, 20, 22, 24, 27]; 221 patients; mean difference of 0.41 mL/kg/min, 95% CI 0.88 to 1.70 mL/kg/min; *I*^2^ = 0%; Figure 5).

**Figure 6:**
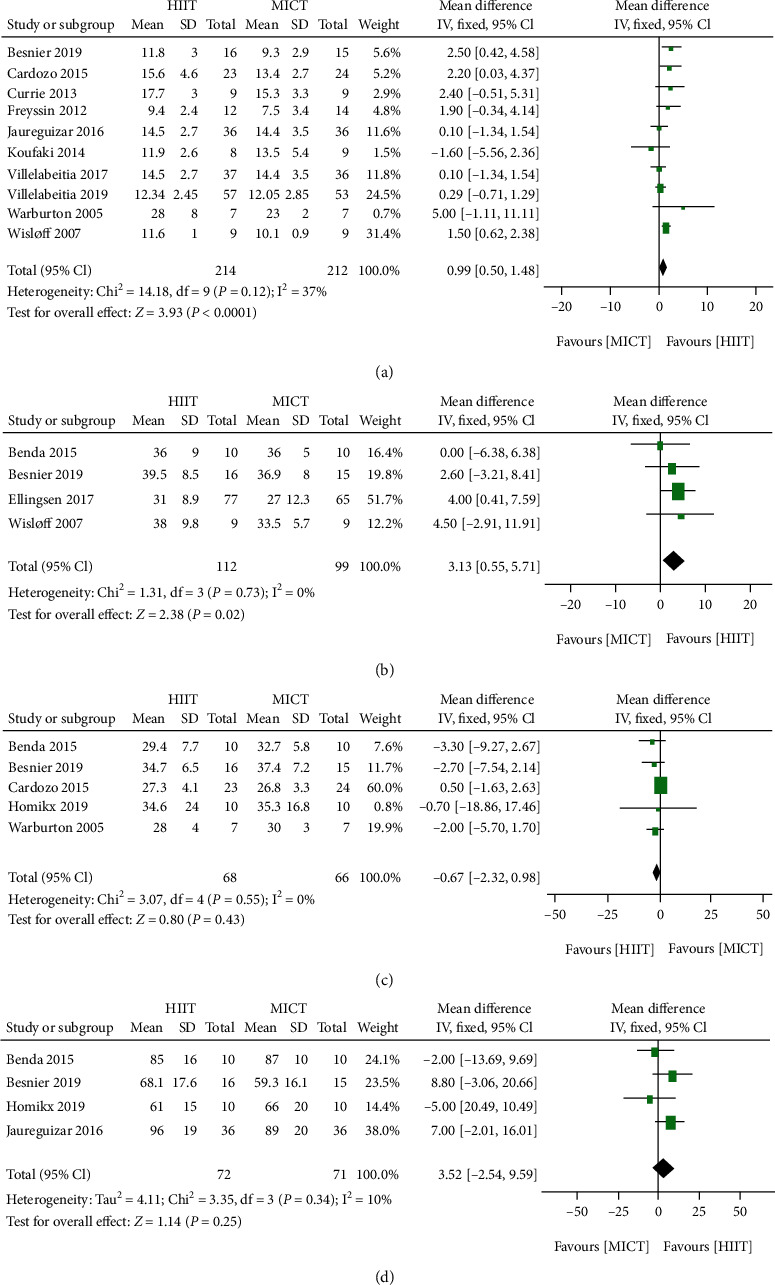
Forest plot summary of the effect of HIIT and MICT on prognostic markers in patients with CAD and HF: (a) anaerobic threshold (AT); (b) left ventricular ejection fraction (LVEF); (c) the VE/carbon dioxide production (VCO_2_) slope (the VE/VCO_2_slope); (d) the prognostic value of percent predicted VO_2_max (the predicted VO_2_ peak (%)). HIIT: high-intensity interval training; MICT: moderate-intensity continuous training; CAD: coronary artery disease; HF: heart failure; SD: standard deviation; CI: confidence interval.

Compared with MICT, HIIT significantly increased the AT (10 trials [16, 18, 20, 23, 25, 26, 28–30]; 446 patients; mean difference of 0.99 mL/kg/min, 95% CI 0.50 to 1.48 mL/kg/min, *P* < 0.0001, *I*^2^ = 37%, *Z* = 3.93; Figure 6(a)) and also caused a moderate increase in LVEF (four trials [16–18, 22]; 211 patients; mean difference of 3.13%, 95% CI 0.55% to 5.71%, *P* = 0.02, *I*^2^ = 0%, *Z* = 2.38; Figure 6(b)). However, HIIT did not have significant effects on the VE/VCO_2_ slope and the predicted VO_2_ peak when compared with MICT (VE/VCO_2_ slope: five trials [17–19, 24, 30]; 132 patients; mean difference of −0.67%, 95% CI −2.32% to 0.98%, *P* = 0.43, *I*^2^ = 0%, *Z* = 0.80. Predicted VO_2_ peak: four trials [17, 18, 24, 25]; 143 patients; mean difference of 3.52%, 95% CI −2.54% to 9.59%, *P* = 0.25, *I*^2^ = 10%, *Z* = 1.14; Figures 6(c) and 6(d)).

## 4. Discussion

This meta-analysis of patients with CAD and HF demonstrated the following three major novel findings. (1) In patients with CAD, HIIT significantly improved the peak VO_2_ value compared with MICT, but no significant difference occurred between HIIT and MICT for patients with HF after subgroup and sensitivity analyses. (2) In patients with CAD and HF, when compared with MICT, HIIT showed that the greatest improvement in the peak VO_2_ value occurs within eight weeks or fewer, but not during weeks 8–12 of training. (3) The increases in the AT and LVEF were significantly higher in the HIIT group than in the MICT group, but there were no significant differences in the VE/VCO_2_ slope and the predicted VO_2_ peak.

In this study, we found that HIIT created a significantly higher peak VO_2_ value in comparison with MICT in patients with CAD and HF. There is a large amount of evidence showing that VO_2_ peak is a powerful independent predictor of total and cardiovascular mortality in patients with cardiovascular disease [31–33], and many previous studies have demonstrated that both MICT and HIIT improve the peak VO_2_ value [34]. The findings in the meta-analyses by Liou et al. [35] indicated that HIIT created a more pronounced, albeit numerically small, improvement in the peak VO_2_ value in patients with stable CAD. Two metastudies comparing the effectiveness of HIIT and MICT on the peak VO_2_ value in patients with heart disease (including CAD and HF) concluded that HIIT appears to be at least as effective as, and in some cases more effective than, MICT [36]. However, in the study conducted by Araújo et al. [37], it was observed that the quality of evidence still does not confirm that HIIT is superior to MICT with respect to the peak VO_2_ value in patients with HF. Our results echoed the findings by Xie et al. [36] that HIIT created a significantly higher peak VO_2_ value in comparison with MICT in patients with CAD and HF, showing that exercise intensity is very important. Smart et al. found that combined strength and intermittent aerobic training exercise appears to be more beneficial for changes in peak VO_2_ values when compared with intermittent aerobic training exercise of similar exercise energy expenditure [38]. However, when we performed subgroup analyses by disease, we found that the advantage of HIIT was primarily reflected in patients with CAD (*P* < 0.0001). Due to the heterogeneity of patients with HF, after sensitivity analyses, the advantage of HIIT on the peak VO_2_ value disappeared (*P* = 0.48); the peak VO_2_ values showed no difference between the groups. This finding is similar to one found in a study by Gomes-Neto et al. [10]; however, it should be interpreted with caution and supported by further research.

Duration may affect the role of HIIT and MICT. Less than eight weeks of HIIT training resulted in higher peak VO_2_ values than MICT for both diseases. In contrast, there were no significant differences in peak VO_2_ improvements in weeks 8–12 of training for both programmes (*P* = 0.53). A study by Ballesta-García et al. [39] drew different conclusions, showing that there were significant differences regarding duration in patients with CAD, with greater improvements in the peak VO_2_ value when the duration was less than 12 weeks. In patients with HF, programmes lasting less than 12 weeks did not significantly improve the peak VO_2_ value. This could be due to a gradual decrease in the effect of HIIT on the improvement of peak VO_2_ values in patients with heart disease as intervention time increases, indicating that the early phase of training is more likely to be responsible for the adaptations of peak VO_2_ values through HIIT. This phenomenon was also detected in highly trained athletes [40]. However, the HIIT programme seems to show advantages when the intervention duration is greater than 12 weeks (*P* = 0.03). This result may be attributed to the fact that only three previous studies [19, 21, 26] have followed a programme for more than 12 weeks. Errors can be introduced into the results due to the lack of research articles, and therefore, caution must be exercised when interpreting these findings.

The major strength of this study is that we performed a subgroup analysis of the peak VO_2_ values, and the results considered in our analysis, such as LVEF, AT, VE/VCO_2_ slope, and predicted VO_2_ peak, are related to the prognosis of patients with heart disease. To our knowledge, this is the first study comparing the prognostic values of these four parameters involved in cardiac disease between two exercise regimens. High-intensity interval training increased the AT by 2.7 mL/kg/min (22%) compared with 1.47 mL/kg/min (12.4%) for MICT. This demonstrates that the AT of HIIT increases more than that of MICT. It has been shown that the AT is associated with oxidation capacity of skeletal muscle, the function of the cardiac pump, and vascular endothelial cells [41]. The AT probably increases the expression of PGC-1 and improves mitochondrial function at the molecular level [16]. In clinical practice, the AT is strongly related to daily activities and quality of life; that is, improving the AT may improve the ability of patients to cope with the physical needs of their daily activities [42].

Our finding that HIIT significantly increased LVEF when compared with MICT is in line with studies by Wisloff et al. [16] and Besnier et al. [18], but in contrast to the meta-analysis of Tucker et al. [43] This result may be associated with the exercise intensity of the interval training, which has been previously reported [40]. In the meta-analysis by Tucker et al. [43], no significant difference in LVEF between HIIT and MICT was detected, which may be related to the relatively low exercise intensity of HIIT participants (87% of the average maximal heart rate (HR_max_) or the peak VO_2_ value) in their four studies compared with the relatively high exercise intensity of MICT participants (70% of the average HR_max_ or the peak VO_2_ value).

The interval training intensity contributes to the functioning of the left ventricle. In experiments conducted by Ellingsen et al. [22], this lower training intensity during intervals becomes more apparent; 51% of the participants in the HIIT group were trained below the regulated intensity, whereas 80% of the participants in the MICT group were trained above the regulated intensity. In contrast, in four studies on LVEF in our meta-analysis, patients in the HIIT group exercised at 89% of their average HR_max_, peak VO_2_ value or max workload and the MICT group exercised at 62% of their average HR_max_, peak VO_2_ value or max workload. Thus, these data suggest that the interval training intensity contributes to the functioning of the left ventricle.

High-intensity interval training may be a more feasible exercise modality for patients with heart disease and can better improve the prognostic effect. Regarding the VE/VCO_2_ slope and the predicted VO_2_ peak aspects, this study found that the magnitude of differences between HIIT and MICT was relatively small, which could be associated with low quality and short-term outcomes in the included studies; this is in accordance with one previous study [36]. Further studies are therefore required to provide more information about the safety and long-term effects of HIIT. Some research has shown that a combination of the VE/VCO_2_ slope and peak VO_2_ value can serve as a more appropriate measure to assess prognosis stratification in patients with HF [44]. The results of our meta-analysis suggested that HIIT may be a more feasible exercise modality for patients with heart disease and can better improve the prognostic effect. In view of improving the VE/VCO_2_ slope and the predicted VO_2_ peak, HIIT was not superior to MICT, and considering the safety and long-term effects of HIIT, exercise-based cardiac rehabilitation programmes should take into account an individual's needs and preferences more often.

There were some limitations in this study. First, only one of the trials in this review used an exercise programme with a 24-week duration, and the others compared the duration of HIIT and MITC in 16 weeks or fewer; consequently, further evidence is required to definitively assess the long-term effects of HIIT on LVEF, AT, the VE/VCO_2_ slope, and the predicted VO_2_ peak. Second, the results of this systematic review may be limited by the lack of clear descriptions of the randomisation and blinding in most studies, as well as the small, pooled sample size (total of 664 adults), with studies ranging from 14 to 142 participants. In addition, the majority of patients with heart disease in this analysis were males (79.9%); therefore, it is unclear that the same results would exist in women with heart disease. The results of this analysis should be interpreted with caution due to the heterogeneity in some subgroup analyses and the small number of trials included.

## 5. Conclusions

For patients with CAD, HIIT is an effective therapy for improving the peak VO_2_ value compared with MICT, while in patients with HF, there is no difference in the improvement in the peak VO_2_ value between the two training methods. During the early stage (eight weeks or fewer), HIIT is superior to MICT. Finally, in heart disease (including CAD and HF), HIIT significantly improved the AT and LVEF compared with MICT, without showing changes in the VE/VCO_2_ slope and the predicted VO_2_ peak. In summary, compared with MICT, HIIT can not only effectively improve the exercise capacity of patients with CAD but also improve the prognosis of patients with CAD and HF.

## Figures and Tables

**Table 1:** Database and search terms.

Database	Search terms	
PubMed	Search ((((“High-Intensity Interval Training”[Mesh]) OR ((((((((((((High Intensity Interval Training[Title/Abstract]) OR High-Intensity Interval Trainings[Title/Abstract]) OR Interval Training, High-Intensity [Title/ Abstract]) OR Interval Trainings, High-Intensity[Title/Abstract]) OR Training, High-Intensity Interval [Title/Abstract]) OR Trainings, High- Intensity Interval[Title/Abstract]) OR High-Intensity Intermittent Exercise [Title/Abstract]) OR Exercise, High-Intensity Intermittent[Title/Abstract]) OR Exercises, High-Intensity Intermittent[Title/Abstract]) OR High- Intensity Intermittent Exercises[Title/Abstract]) OR Sprint Interval Training[Title/Abstract]) OR Sprint Interval Trainings[Title/Abstract]))) AND (((((((Cardiac Rehabilitations[Title/Abstract]) OR Rehabilitation, Cardiac[Title/Abstract]) OR Rehabilitations, Cardiac[Title/Abstract]) OR Cardiovascular Rehabilitation[Title/Abstract]) OR Cardiovascular Rehabilitations[Title/Abstract]) OR Rehabilitation, Cardiovascular [Title/ Abstract]) OR Rehabilitations, Cardiovascular[Title/Abstract])) AND ((randomized controlled trial[Publication Type] OR randomized [Title/ Abstract] OR placebo[Title/Abstract]))	
Embase	Session results	
	No. query results	Result date
	#24. #1 AND #10 AND #23	1 3 Dec 2019
	#23. #11 OR #12 OR #13 OR #14 OR #15 OR #16 OR #17 OR	2,178 3 Dec 2019
	#18 OR #19 OR #20 OR #21 OR #22	
	#22. ‘sprint interval trainings':ti,ab	1 3 Dec 2019
	#21. ‘sprint interval training':ti,ab	250 3 Dec 2019
	#20. ‘high-intensity intermittent exercises':ti,ab	12 3 Dec 2019
	#19. ‘exercises, high-intensity intermittent':ti,ab	3 Dec 2019
	#18. ‘exercise, high-intensity intermittent':ti,ab	2 3 Dec 2019
	#17. ‘high-intensity intermittent exercise':ti,ab	242 3 Dec 2019
	#16. ‘trainings, high-intensity interval':ti,ab	3 Dec 2019
	#15. ‘training, high-intensity interval':ti,ab	11 3 Dec 2019
	#14. ‘interval trainings, high-intensity':ti,ab	3 Dec 2019
	#13. ‘interval training, high-intensity':ti,ab	1 3 Dec 2019
	#12. ‘high-intensity interval trainings':ti,ab	3 3 Dec 2019
	#11. ‘high intensity interval training'/exp	1,831 3 Dec 2019
	#10. #2 OR #3 OR #4 OR #5 OR #6 OR #7 OR #8 OR #9	11,281 3 Dec 2019
	#9. ‘rehabilitations, cardiovascular':ti,ab	3 Dec 2019
	#8. ‘rehabilitation, cardiovascular':ti,ab	8 3 Dec 2019
	#7. ‘cardiovascular rehabilitations':ti,ab	3 Dec 2019
	#6. ‘cardiovascular rehabilitation':ti,ab	511 3 Dec 2019
	#5. ‘rehabilitations, cardiac':ti,ab	3 Dec 2019
	#4. ‘rehabilitation, cardiac':ti,ab	37 3 Dec 2019
	#3. ‘cardiac rehabilitations':ti,ab	10 3 Dec 2019
	#2. ‘heart rehabilitation'/exp	10,958 3 Dec 2019
	#1. ‘'random':ab,ti OR ‘placebo':ab,ti OR	658,790 3 Dec 2019
	‘'double-blind':ab,ti	
Cochrane	Search name: Cochrane-OA Last saved:03/12/2019 23:23:03 Comment: ID search #1MeSH descriptor: [Cardiac Rehabilitation] explode all trees #2(Cardiac Rehabilitations):ti,ab,kw OR (Rehabilitation, Cardiac):ti,ab,kw OR (Rehabilitations, Cardiac):ti,ab,kw OR (Cardiovascular Rehabilitation):ti,ab,kw OR (Cardiovascular Rehabilitations):ti,ab,kw (Word variations have been searched) #3(Rehabilitation, Cardiovascular):ti,ab,kw OR (Rehabilitations, Cardiovascular):ti,ab,kw (Word variations have been searched) #4#1 or #2 or #3 #5MeSH descriptor: [High-Intensity Interval Training] explode all trees #6(High-Intensity Interval Trainings):ti,ab,kw OR (Interval Training, High-Intensity):ti,ab,kw OR (Interval Trainings, High-Intensity):ti,ab,kw OR (Training, High-Intensity Interval):ti,ab,kw OR (Trainings, High-Intensity Interval):ti,ab,kw (Word variations have been searched) #7(High-Intensity Intermittent Exercise):ti,ab,kw OR (Exercise, High-Intensity Intermittent):ti,ab,kw OR (Exercises, High-Intensity Intermittent):ti,ab,kw OR (High-Intensity Intermittent Exercises):ti,ab,kw OR (Sprint Interval Training):ti,ab,kw (Word variations have been searched) #8(Sprint Interval Trainings):ti,ab,kw (Word variations have been searched) #9#5 or #6 or #7 #8 #10#4 AND #9	
Web of Science	#1 TS=(Cardiac Rehabilitation OR Cardiac Rehabilitation∗ OR Rehabilitation, Cardiac OR Rehabilitation∗, Cardiac OR Cardiovascular Rehabilitation OR Cardiovascular Rehabilitation∗ OR Rehabilitation, Cardiovascular OR Rehabilitation∗, Cardiovascular) #2 TS=(High Intensity Interval Training OR High-Intensity Interval Training∗ OR Interval Training, High-Intensity OR Interval Training∗, High-Intensity OR Interval Training∗, High-Intensity OR Interval Training∗, High-Intensity OR Training, High-Intensity Interval OR Training∗, High-Intensity Interval OR High-Intensity Intermittent Exercise OR Exercise, High-Intensity Intermittent OR Exercises, High-Intensity Intermittent OR High-Intensity Intermittent Exercise∗ OR Sprint Interval Training OR Sprint Interval Training∗) #3 TS=(random∗ controlled trial OR random∗OR placebo) #4 #3 AND #2 AND #1	
ClinicalTrials	Search “High-Intensity Interval Training” AND “Cardiac Rehabilitations”	
Wanfang Database	“心脏康复”和“高强度间歇训练”	
China National Knowledge Internet	“心脏康复”和“高强度间歇训练”	
CBM	“心脏康复”和“高强度间歇训练”	

**Table 2:** General characteristics of each study included in the meta-analysis.

Study, year	Disease	HIIT					MICT				
		Sample size	Gender	Age (years)	BMI (kg m^−2^)	LVEF (%)	Sample size	Gender	Age (years)	BMI (kg m^−2^)	LVEF (%)
Benda 2015 [26]	HF	10	9 M; 1 F	63 ± 8	28.1 ± 7.5	37 ± 6	10	10 M	64 ± 8	28.9 ± 4.7	38 ± 6
Besnier 2019 [33]	HF	16	11 M; 5 F	59 ± 13	25 ± 5	36 ± 8	15	14 M; 4 F	59.5 ± 12	28 ± 5	36 ± 7
Cardozo 2015 [27]	CAD	23	14 M; 9 F	56 ± 12	27.5 ± 5.9	60 ± 14	24	16 M; 8 F	62 ± 12	26.8 ± 4.8	63 ± 12
Currie 2013 [32]	CAD	11	NA	62 ± 11	27.9 ± 4.9	NA	11	NA	68 ± 8	27.3 ± 4.2	NA
Currie 2015 [31]	CAD	9	9 M	63 ± 8	28.9 ± 4.8	NA	10	9 M; 1 F	66 ± 8	27.3 ± 4.0	NA
Ellingsen 2017 [29]	HF	77	63 M; 14 F	65 ± 22.4	27.6 ± 5.4	29 ± 11.19	65	53 M; 12 F	60 ± 14.4	27.5 ± 6.4	29 ± 12.4
Freyssin 2012 [28]	HF	12	6 M; 6 F	54 ± 9	24.8 ± 4.0	27.8 ± 4.7	14	7 M; 7 F	55 ± 12	24.1 ± 5.4	30.7 ± 7.8
Hornikx 2019 [30]	HF	10	5 M; 5 F	64 ± 8	26 ± 4	30 ± 14	10	6 M; 4 F	58 ± 11	29 ± 4	31 ± 14
Jaureguizar 2016 [35]	CAD	36	28 M; 8 F	58 ± 11	29.6 ± 4.6	62 ± 11	36	33 M; 3 F	58 ± 11	29.5 ± 4.1	59 ± 14
Koufaki 2014 [10]	HF	16	14 M; 2 F	59.8 ± 7.4	28.9 ± 4.7	41.7 ± 10.3	17	13 M; 4 F	59.7 ± 10.8	29.5 ± 4.7	35.2 ± 6.4
Rognmo 2004 [34]	CAD	8	6 M; 2 F	62.9 ± 11.2	26.7 ± 4.1	54.8 ± 9.1	9	8 M; 1 F	61.2 ± 7.3	26.9 ± 2.7	51.9 ± 9.6
Villelabeitia 2017 [36]	CAD	37	29 M; 8 F	58 ± 11	29.6 ± 4.4	62 ± 11	36	33 M; 3 F	58 ± 11	29.5 ± 4.1	59 ± 14
Villelabeitia 2019 [37]	CAD	57	50 M; 7 F	57.6 ± 9.8	29.1 ± 3.9	61.2 ± 10.1	53	42 M; 11 F	58.3 ± 9.5	27.8 ± 3.7	60.3 ± 9.7
Warburton 2005 [38]	CAD	7	NA	55 ± 7	NA	NA	7	NA	57 ± 8	NA	NA
Wisløff 2007 [16]	HF	9	7 M; 2 F	76.5 ± 9	24.5 ± 3	28.0 ± 7.3	9	7 M; 2 F	74.4 ± 12	24.7 ± 3	32.8 ± 4.8

**Table 3:** Characteristics for HIIT and MICT interventions.

Study	Duration (weeks)	Exercise modality	HIIT				MICT			
			Exercise intensity (%max) (interval: rest)	Frequency (days/week)	Exercise time per week (min)	Attendance rate, dropouts and adverse events	Exercise intensity (%max) (interval: rest)	Frequency (days/week)	Exercise time per week (min)	Attendance rate, dropouts and adverse events
Benda	12	Cycle	10 × 1 min90% Maximal workload: 10 × 2.5 min30% Maximal workload	2	70	Attendance NR; dropouts = 2 (16%); adverse: NR	60-75% Maximal workload	2	60	Attendance NR; dropouts = 2 (16%); adverse: NR
Besnier	3.5	Cycle	80% PPO	5	150	Attendance = 94%; dropouts = 1; adverse = 0	60% PPO	5	150	Attendance: 100% Dropouts = 1; adverse = 0
Cardozo	16	Running	2 min 90% HR_max_: 2 min 60% HR_max_	3	120	Attendance: NR; dropouts: NR; adverse = 0	70-75%HR_max_	3	120	Attendance: NR; dropouts: NR; adverse = 0
Currie	12	Cycle	2 × 8 min100% PPO (30s traning, 30 s rest)	2	80	Attendance: NR; dropouts: NR Adverse = 0	58% PPO	2	80-100	Attendance: NR; dropouts: NR; adverse = 0
Currie	24	Cycle	121% PPO	2	120	Attendance: NR; dropouts = 1 Adverse = 1	78% PPO	2	120	Attendance: NR Dropouts = 2; adverse = 2
Ellingsen	12	Running or cycle	4 × 4 min 90-95%HR_max_; 3 min 60-70% HR_max_	NR	NR	Attendance = 94%; dropouts = 1; adverse = 2	60-70%HR_max_	NR	NR	Attendance = 94%; dropouts = 3; adverse = 4
Freyssin	8	Running or cycle	80% maximal power	5	168	Attendance = 100%; dropouts = 0; adverse = 0	50% maximal power	5	360	Attendance = 100%; dropouts = 0; adverse = 0
Hornikx	12	Running or cycle	5 × 3 min 80% Wpeak; 4 × 3 min 40% Wpeak	3	99	Attendance: NR; dropouts = 1 (10%); adverse = 1 (10%)	50%Wpeak	3	180	Attendance: NR; dropouts = 1 (10%); adverse = 3 (33%)
Jaureguizar	8	Cycle	105-135% VO_2peak_	3	120	Attendance = 92%; dropouts = 0; adverse = 0	65-70% VO_2peak_	3	120	Attendance = 87.5%; dropouts = 0; adverse = 0
Koufaki	24	Cycle	100% VO_2peak_	3	120	Attendance = 94%; dropouts = 1 (6%); adverse = 1 (6%)	40-60% VO_2peak_	3	120	Attendance = 100%; dropouts = 3; adverse = 3
Rognmo	10	Running	4 × 4 min 80-90% VO_2peak_; 50-60% VO_2peak_	3	99	Attendance = 77% Dropouts = 3; adverse = 0	50-60% VO_2peak_	3	123	Attendance = 90%; dropouts = 1; adverse = 0
Villelabeitia	8	Cycle	105-135% VO_2peak_	3	120	Attendance = 92%; dropouts = 0; adverse = 0	65-70% VO_2peak_	3	120	Attendance = 87.5%; dropouts = 0; adverse = 0
Villelabeitia	8	Cycle	108-126% VO_2peak_	3	120	Attendance = NR; dropouts = 0; adverse = 0	60-70% VO_2peak_	3	120	Attendance = NR Dropouts = 0; adverse = 0
Warburton	16	Running or climb stairs	90% heart rate/VO2 reserve	2	60	Attendance: NR; dropouts = 0; adverse: NR	65% heart rate/VO2 Reserve	2	60	Attendance: NR; dropouts = 0; adverse: NR
Wisløff	12	Running	4 × 4 min 90-95% VO2peak; 50-60% VO2peak	3	114	Attendance = 92%; dropouts = 0; adverse = 0	70-75% VO2peak	3	141	Attendance = 95%; dropouts = 1; adverse = 0

HIIT: high-intensity interval training; MICT: moderate-intensity continuous training; PPO: peak power output; HR_max_: maximal heart rate; Wpeak: peak workload; VO_2peak_: peak oxygen uptake; NR: not reported.

## Data Availability

The datasets used and analyzed during the current study are available from the corresponding author on reasonable request.
